# Classification of Tidal Breathing Airflow Profiles Using Statistical Hierarchal Cluster Analysis in Idiopathic Pulmonary Fibrosis

**DOI:** 10.3390/medsci6030075

**Published:** 2018-09-12

**Authors:** E. Mark Williams, Ricardo Colasanti, Kasope Wolffs, Paul Thomas, Ben Hope-Gill

**Affiliations:** 1 Faculty of Life Sciences and Education, University of South Wales, Pontypridd CF37 1DL, UK; 2 Department of Computer Science, Swansea University, Swansea SA2 8PP, UK; r.l.colasanti@swansea.ac.uk; 3 Cardiff School of Biosciences, Cardiff University, Cardiff CF10 3AT, UK; olukogbonkl2@cardiff.ac.uk; 4 Lung Function Laboratory, University Hospital Llandough, Llandough CF64 2XX, UK; paul.thomas7@wales.nhs.uk; 5 Department of Respiratory Medicine, University Hospital Llandough, Llandough CF64 2XX, UK; Ben.hope-gill@wales.nhs.uk

**Keywords:** euclidian distance, minute ventilation, tidal volume, unstructured learning, lung function, inspiratory expiratory time

## Abstract

In idiopathic pulmonary fibrosis (IPF) breathing pattern changes with disease progress. This study aims to determine if unsupervised hierarchal cluster analysis (HCA) can be used to define airflow profile differences in people with and without IPF. This was tested using 31 patients with IPF and 17 matched healthy controls, all of whom had their lung function assessed using spirometry and carbon monoxide CO transfer. A resting tidal breathing (RTB) trace of two minutes duration was collected at the same time. A Euclidian distance technique was used to perform HCA on the airflow data. Four distinct clusters were found, with the majority (18 of 21, 86%) of the severest IPF participants (Stage 2 and 3) being in two clusters. The participants in these clusters exhibited a distinct minute ventilation (*p* < 0.05), compared to the other two clusters. The respiratory drive was greatest in Cluster 1, which contained many of the IPF participants. Unstructured HCA was successful in recognising different airflow profiles, clustering according to differences in flow rather than time. HCA showed that there is an overlap in tidal airflow profiles between healthy RTB and those with IPF. The further application of HCA in recognising other respiratory disease is discussed.

## 1. Introduction

The way we breathe is influenced by several factors, such as lung mechanics, neurological drive, and emotional status [1]. Changes in lung ventilation are achieved via changes in breathing rate, regularity, and depth [2]. In the presence of lung disease when lung mechanics are altered, the airflow profile is also altered [3]. In obstructive lung disease, such as chronic obstructive disease (COPD) and cystic fibrosis, the change in expired airflow profile is linked to the severity of the airway obstruction [4,5,6,7]. In idiopathic pulmonary fibrosis (IPF), a largely restrictive disease, tidal breathing is altered via an increase in minute volume, ${\dot{V}}_{E}$, with the increase in ${\dot{V}}_{E}$ being met by an increased tidal volume rather than breathing rate [8,9]. In IPF, the airflow flow profile is also characterised by an increase in peak inspired and expired flow [9]. These parameters provide an insight into how breathing changes with disease but fail to provide unique time or flow characteristics of the airflow signal.

The aim of this study is to use unsupervised hierarchal cluster analysis on data sets of tidal breathing airflow profiles in people with and without IPF. The advantages of these data mining methods are that they can distinguish any post-priori patterns in the airflow profiles. This is in comparison to supervised learning techniques such as naive Bayesian and decision tree techniques, which assume a priori classification of the data and identification of its key attributes [10]. The clusters of patterns defined by hierarchal cluster analysis once identified can be compared for their biological characteristics. Cluster analysis has been used to identify phenotypes in asthma, but this is the first time it has been applied to respiratory breathing patterns [11].

## 2. Methods

### 2.1. Subjects

Thirty-one patients with a diagnosis of IPF attending the Cardiff Interstitial Lung Diseases Clinic and 17 healthy, age-matched, non-smoking controls were recruited for a study investigating breathing patterns [9]. The study was approved by the South East Wales Regional Ethics Committee (REC reference number: 13/WA/0200). The Faculty of Life Sciences and Education ethics committee, University of South Wales, approved the protocol for the control group. Informed written consent was provided by all participants in the study.

### 2.2. Pulmonary Function Testing

The tidal breathing and pulmonary function tests were performed using Jaeger Masterscreen Systems PFT suite (Carefusion, UK). All tests were performed with the subject seated whilst wearing nose-clips. Whilst breathing for two minutes through a mouthpiece, connected in series to a bacterial filter and pneumotach, tidal airflow was collected (at 100 Hz).

The severity of the IPF was categorised into three stages (1: mild, *n* = 10; 2: moderate *n* = 12; 3: severe, *n* = 9) based on gender (G), age (A), and physiological variables (P) (Forced Vital Capacity %predicted and TLCO %predicted) using the GAP index [9,12].

### 2.3. Hierarchal Cluster Analysis

From each participant’s tidal breathing recording, the last 10% and first 20% were removed, and the reaming recording was conditioned and smoothed using a running average of 200 ms, enabling each breath to be defined and isolated. The extraordinary breaths, either larger or smaller that fell outside one standard deviation of the recording being analysed were discarded. Each of the remaining breaths were normalised by time and a single mean breath was then derived for each participant (*n* = 48); these single breaths were used in all further analyses. 

**Figure 1:**
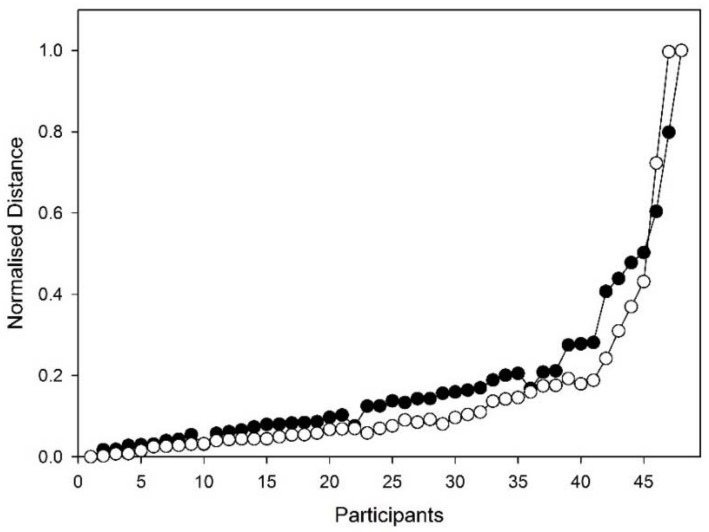
The number-distance plots of the Euclidian (●) and Pearson (○) methods.

Hierarchal analysis began with each breath being compared to all other breaths. In each case, the same time point was used. This produced a 30 × 30 data matrix. Two matrices were created using a Euclidian distance technique and Pearson correlation [13]; two further matrices were created using breath data that was normalised for time and amplitude. The hierarchical clustering methods were compared using a number-distance plot (Figure 1), which suggested that time only normalised Euclidian clustering was the best method. 

The programme script for data conditioning was written in Python using the SCIPY library [14] and the algorithms performing the Euclidian and Pearson’s analysis along with the hierarchal clustering analysis were written by RC.

### 2.4. Statistical Analysis

Data were expressed as means and standard deviations for normally distributed data and the median plus the range for non-normally distributed parameters. Differences between clusters were tested by analysis of variance, with post-hoc analysis where appropriate. Linear correlations were assessed by linear regression. Statistical significance is defined when *p* < 0.05. All statistical analyses were performed using Sigmaplot v14 (Systat Software Inc., London, UK).

## 3. Results

**Figure 2:**
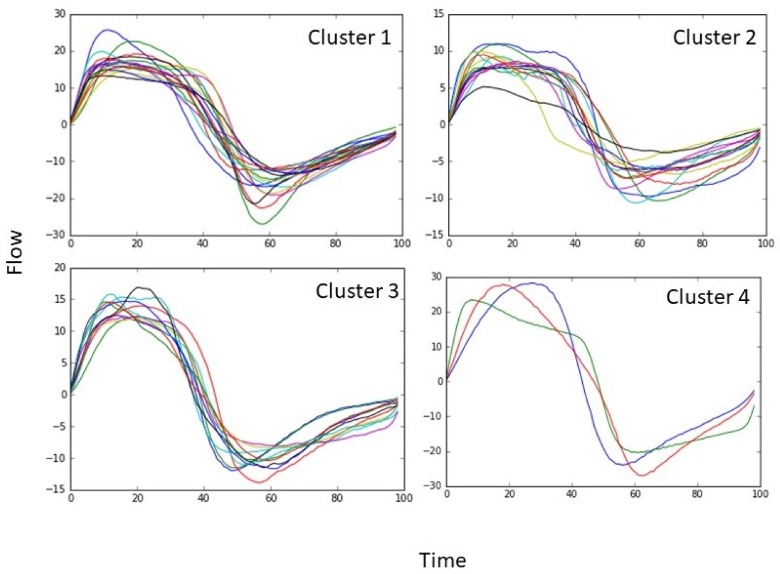
Mean breaths for each participant. Normalised for time (*X*-axis) and flow (*Y*-axis), the four clusters.

**Figure 3:**
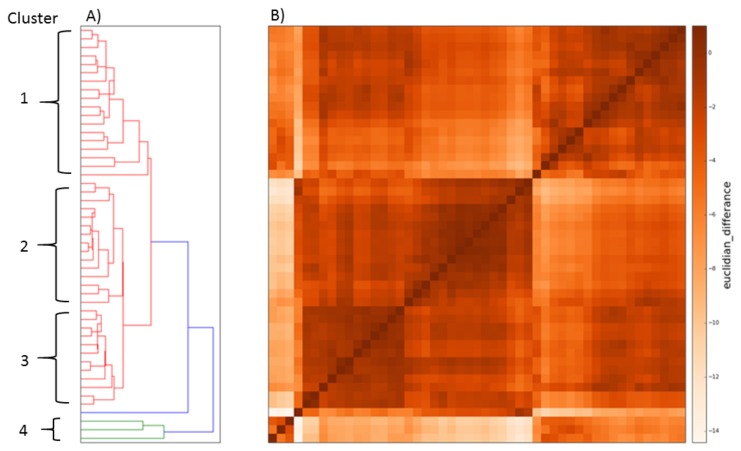
Clusters defined from (**A**) dendrogram and (**B**) heat map following cluster analysis. The single trace was excluded from the numerical analysis due to missing data.

**Figure 4:**
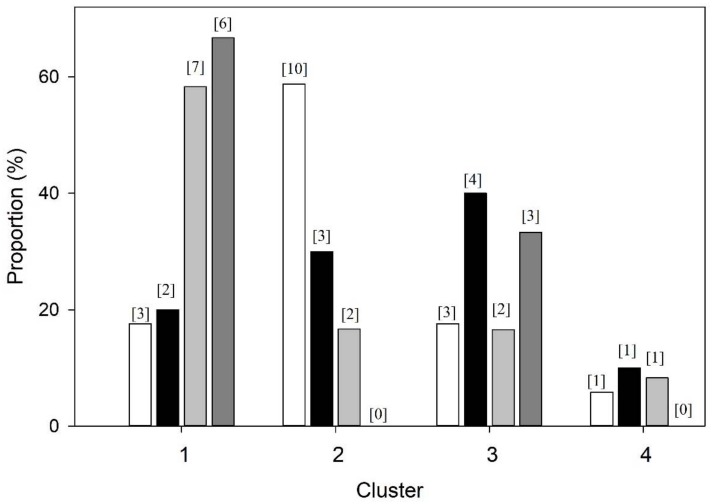
Percentage distribution of participants (three idiopathic pulmonary fibrosis (IPF) groups and controls) within clusters. Controls (white), *n* = 18; IPF Stage 1 (black), *n* = 10; IPF Stage 2 (light grey), *n* = 12; IPF Stage 3 (grey), *n* = 9.

The time-normalised mean breath was analysed for all participants (*n* = 48) irrespective of disease status or health. Euclidean distance cluster analysis (EDCA) grouped the data set into four distinct clusters characterized in Table 1 (Figure 2, Figure 3 and Figure 4). Breaths from the controls appear in all four cluster groups, but the majority (59%) were found in Cluster 2, whereas those IPF patients with Stage 3 disease were only found in only two clusters, with two-thirds (67%) being classified in Cluster 1 and the remainder in Cluster 3 (Figure 2 and Figure 4). Clusters 2 and 4 consisted of controls as well as Stage 1 and 2 patients (Figure 2 and Figure 4).

**Figure 5:**
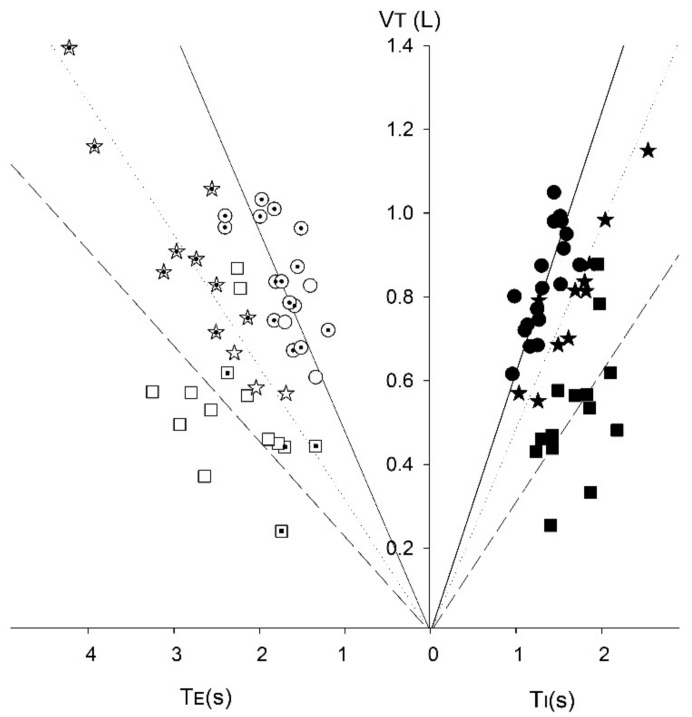
VT-T_I_-T_E_ diagram for each cluster group: Cluster 1 (●), Cluster 2 (■), Cluster 3 (✯), Cluster 4 not shown. The filled T_E_ symbols denote IPF participants. Fitted linear regression lines shown.

A statistical comparison of the non-normalised data between clusters using ANOVA (Table 1) show differences in timing indices and flow characteristics (*p* < 0.05) (Table 1). The largest cluster (Cluster 1, *n* = 18) had a high ${\dot{V}}_{E}$, 16.5 ± 2.0 L min^−1^ (mean ± SD), resulting from a rapid breathing rate, 20 ± 3 breaths min^−1^, and tidal volume, V_T_ 0.83 L (0.62–1.05) (median (range)). Cluster 2 (*n* = 14) was characterized by a low ${\dot{V}}_{E}$ of 8.1 ± 2.3 L min^−1^ consisting of a breathing rate of 16 ± 3 breaths min^−1^ and V_T_ of 0.51(0.26–0.88). Cluster 3 (*n* = 12) had a higher ${\dot{V}}_{E}$ than Cluster 2 but lower than Cluster 1, at 11.4 ± 1.3 L min^−1^, achieved by a low breathing rate, 14 ± 4 breaths min^−1^, and high V_T_ 0.81 L (0.55–1.45). Cluster 4 (*n* = 3) had the highest ${\dot{V}}_{E}$, 25.7 ± 1.3 L min^−1^, and was characterized by a high breathing rate of 26 ± 3 breaths min^−1^, and V_T_, 1.04 L (0.84–1.07). Further analysis shows that each group has its own distinct respiratory drive, as indicated by the differing slope of the isoflow lines (Figure 5) [15]. Cluster 1 (high ${\dot{V}}_{E}$), exhibiting the strongest drive, indicated by the steepest isoflow line, whilst Cluster 2 (low ${\dot{V}}_{E}$) exhibited the lowest drive.

**Figure 6:**
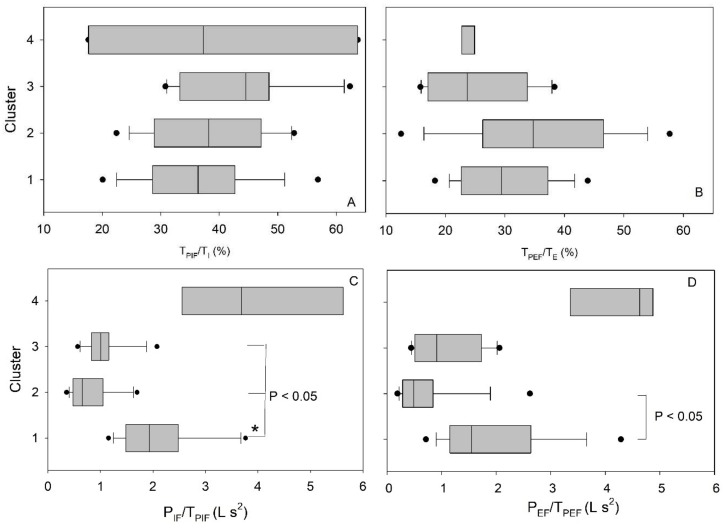
(**A**) T_PIF_/T_I_, (**B**) T_PEF_/T_E_, (**C**) P_IF_/T_PIF_, (**D**) P_EF_/T_PEF_. Cluster 1 was significantly different from Cluster 2. No significant differences were found (*p* > 0.05), except where indicated.

Differences in the relationship between peak inspiratory and expiratory flow and group were observed (Figure 6). The rate to reach maximum flow, P_IF_/T_PIF_ and P_EF_/T_PEF_, (P_IF_: peak inspiratory flow, P_EF_: Peak expiratory flow, T_PIF_: time to peak inspiratory flow, T_PEF_: time to peak expiratory flow) was lengthened in Cluster 1 (high ${\dot{V}}_{E}$) only. An analysis of the clinical parameters between the clusters show that the FVC % predicted, FEV_1_/FVC ratio, and TLCO % predicted were different (Table 2). Cluster 1 (high ${\dot{V}}_{E}$) exhibited values akin to diminished lung function (Table 2). The IPF Stage 3 patients, clustered into Cluster 1 (high ${\dot{V}}_{E}$) and 3 (low Bf), had different PaCO_2_ partial pressures at 4.6 ± 0.5 and 5.2 ± 0.5 kPa, respectively (*p* = 0.015), whilst arterial PO_2_, O_2_ saturation, and cough were not different (*p* < 0.05).

## 4. Discussion

Undirected statistical hierarchal cluster (USHC) analysis of the resting tidal breathing airflow profiles recorded from patients with IPF and age-similar controls revealed four distinct clusters: three major and one minor cluster (Figure 2). Each cluster consisted of a mixture of both patients and controls, with cluster membership unrelated specifically to disease status or symptoms (Table 1 and Table 2). Statistical analysis of each cluster’s characteristics showed that clustering was based on differences in ventilation rate, ${\dot{V}}_{E}$. However, the distribution of patients and controls across the cluster does suggest that most of the patients with Stage 2 and 3 IPF had altered breathing patterns, with an intermittent ${\dot{V}}_{E}$ (in Cluster 1 and 3).

The use of a single representative (normalised) breath for each participant, rather than analysing multiple breaths, was important for pattern recognition. When recording a series of breaths, there is always breath-to-breath variation in duration and magnitude of each breath, a phenomenon determined largely by neural drive. Time normalisation removes this variability, and thus any differences remaining in the airflow profile result from altered mechanical function. Normalisation was successfully used in the previous studies, where time and flow indices were used to define disease status [6,7]. 

USHC analysis without a priori selection of any flow, time, disease, or symptom characteristics allows any intrinsic profile phenomenon associated with breathing mechanics to be isolated. The four clusters are characterised by their ventilation patterns, Cluster 1 (*n* = 18) has a high ${\dot{V}}_{E}$, which results in this case from both a fast breathing rate and raised VT, which is unlike the IPF group alone (Table 1) [9]. This is the largest group and consists of largely participants with IPF (83%) and does illustrate that IPF does alter breathing patterns, thus supporting the conclusion that minute ventilation is raised in this group [9]. Cluster 2 (*n* = 14), the second largest group, is characterised by a low ${\dot{V}}_{E}$, reflecting a breathing rate and VT range seen in healthy subjects, and consists principally of control participants (67% of the cluster). Cluster 3 (*n* = 12) has an intermediate ${\dot{V}}_{E}$, again composed of a slow breathing rate but countered by a high VT. This cluster has a mixture of participants; the hypoventilatory features apparent in this cluster are reflected by the Class 3 IPF participants who have a significantly raised PaCO_2_ (in comparison to Cluster 1). The forth cluster (*n* = 3) is characterised by hyperventilation, having a high ${\dot{V}}_{E}$, high breathing rate, and high tidal volume (Table 1). Overall, the analysis shows that the IPF-free participants breathed using a variety of patterns, whereas those with IPF were less variable. Tracking this loss of variability with disease progression via a longitudinal study may prove to have some prognostic value.

The clustering by ventilation is further exemplified by the V_T_/T_I_-T_E_ relationship, with Cluster 1 showing the steepest relationship between these parameters (Figure 5) and consisting mainly of IPF participants. The steeper gradient of the V_T_/T_I_ and V_T_/T_E_ relationship in Cluster 1 also implies that the maximum ventilation rate, V_max_, would be reached sooner in exercise [15]. Thus, resting breathing patterns in people with IPF (and Cluster 1 controls) may be a predictor for exercise intolerance, especially if this were linked to PaCO_2_ and arterial O_2_ saturation levels.

Defining resting tidal breathing via its shape time profile using cluster analysis provides a different view than using specific volumetric/flow parameters used in previous studies [6,7]. The mixing of patterns between those with healthy and fibrotic lungs illustrates that resting tidal breathing (RTB) patterns are complex and not just based on respiratory mechanics. 

### Limitations

This study is limited by using a small diverse group of participants, including a clinical group of varying severity. Larger participant numbers across all health and IPF classifications would better inform the cluster analysis, which might then find more than four clusters, or may even better define the IPF stages and controls. 

## 5. Conclusions

Hierarchal cluster analysis using Euclidian distance defined four distinct clusters, which are characterised by their different ventilation rates rather than disease status, which illustrates that breathing pattern generation is influenced by a range of biological factors, separate from lung function. However, although clustering was observed to provide some degree of selectivity for disease or healthy subjects, further analysis of larger groups and different lung disease groups are required before the full potential of hierarchal cluster analysis of resting breathing airflow profiles is realised.

## Figures and Tables

**Table 1:** Comparisons between breathing parameters in cluster groups.

	Cluster 1 *n* = 18	Cluster 2 *n* = 14*	Cluster 3 *n* = 12	Cluster 4 *n* = 3	*p*-Value
T_tot_ (s)	3.06 ± 0.50	3.96 ± 0.77	4.48 ± 1.22	2.30 ± 0.23	*p* < 0.001 ^a,b,e,f^
T_I_ (s)	1.34 ± 0.22	1.69 ± 0.31	1.76 ± 0.49	1.07 ± 0.18	*p* < 0.001 ^a,b,d,f^
T_E_ (s)	1.72 ± 0.32	2.26 ± 0.54	2.73 ± 0.75	1.23 ± 0.06	*p* < 0.001 ^a,b,e,f^
Breathing rate (breaths min^−1^)	20 ± 3	16 ± 3	14 ± 4	26 ± 3	*p* < 0.001 ^a,b,c,e,f^
T_i_/T_tot_ (range)	0.44 (0.37–0.49)	0.43 (0.31–0.49)	0.39 (0.37–0.44)	0.47 (0.43–0.49)	*p* < 0.001 ^b,d,f^
V_E_ (L min^−1^)	16.5 ± 2.0	8.1 ± 2.3	11.4 ± 1.3	25.7 ± 1.3	*p* < 0.001 ^a,b,c,d,e,f^
P_IF_ (L s^−1^)	0.90 (0.69–1.31)	0.47 (0.24–0.60)	0.72 (0.63–0.89)	1.44 (1.23–1.48)	*p* < 0.001 ^a,d,e^
P_EF_ (L s^−1^)	0.80 (0.64–1.48)	0.36 (0.22–0.56)	0.60 (0.35–1.56)	1.23 (1.07–1.48)	*p* < 0.001 ^a,b,e,f^
T_PIF_ (s)	0.48 (0.22–0.74)	0.60 (0.33–1.02)	0.72 (0.36–1.57)	0.40 (0.22–0.57)	*p* < 0.005
T_PEF_ (s)	0.52 (0.29–1.05)	0.88 (0.21–1.28)	0.63 (0.33–1.56)	0.31 (0.27–0.32)	0.008 ^e^
V_Tin_ (L)	0.83 (0.62–1.05)	0.51(0.26–0.88)	0.81 (0.55–1.45)	1.04 (0.84–1.07)	*p* < 0.001 ^a,d,e^
V_Tout_ (L)	0.84 ± 0.13	0.53 ± 0.16	0.87 ± 0.24	0.96 ± 0.11	*p* < 0.001 ^a,d,e^

The mean standard seviation (SD) is shown or the median and range as indicated. TTOT: duration of breath, TI: Inspiratory time, TE: expiratory time, PIF: peak inspiratory flow, PEF: peak expiratory flow, TPIF: time to peak inspiratory flow, TPEF: time to peak expiratory flow. Total *n* = 47, one case was unusable. ANOVA or ANOVA with ranks if not normally distributed. Post hoc testing (Holm-Sidak and Dunn’s Method) allowed multiple comparisons between clusters with differences indicated by superscripts, ^a^ Cluster 1 and 2, ^b^ Cluster 1 and 3, ^c^ Cluster 1 and 4, ^d^ Cluster 2 and 3, ^e^ Cluster 2 and 4, ^f^ Cluster 3 and 4.

**Table 2:** Comparison of lung function parameters in cluster groups

	Cluster 1 *n* = 18	Cluster 2 *n* = 15	Cluster 3 *n* = 12	Cluster 4 *n* = 3	*p*-Value
FVC % predicted	80 ±24	109 ± 22 *	102 ± 36	102 ± 10	*p* = 0.022
FEV_1_/FVC (%)	82 ±7	78 ± 7	74 ± 9 *	75 ± 6	*p* = 0.026
TLCO % predicted	30 (17–111)	75 (33–95) *	59 (30–92)	74 (48–91)	*p* = 0020

Mean ± SD, or Median (range) shown. * Different to Cluster 1. See key Table 1.
